# Effects of variety and nutrient availability on the acrylamide-forming potential of rye grain

**DOI:** 10.1016/j.jcs.2013.02.001

**Published:** 2013-05

**Authors:** Jennifer Postles, Stephen J. Powers, J. Stephen Elmore, Donald S. Mottram, Nigel G. Halford

**Affiliations:** a Plant Biology and Crop Science Department, Rothamsted Research, Harpenden, Hertfordshire AL5 2JQ, United Kingdom; b Computational and Systems Biology Department, Rothamsted Research, Harpenden, Hertfordshire AL5 2JQ, United Kingdom; c Department of Food and Nutritional Sciences, University of Reading, Whiteknights, Reading RG6 6AP, United Kingdom

**Keywords:** *Secale cereale*, Process contaminant, Asparagine, Sugars

## Abstract

Acrylamide is a probable human carcinogen that forms in plant-derived foods when free asparagine and reducing sugars react at high temperatures. The identification of rye varieties with low acrylamide-forming potential or agronomic conditions that produce raw material with low acrylamide precursor concentrations would reduce the acrylamide formed in baked rye foods without the need for additives or potentially costly changes to processes. This work compared five commercial rye varieties grown under a range of fertilisation regimes to investigate the effects of genotype and nutrient (nitrogen and sulphur) availability on the accumulation of acrylamide precursors. A strong correlation was established between the free asparagine concentration of grain and the acrylamide formed upon heating. The five rye varieties accumulated different concentrations of free asparagine in the grain, indicating that there is genetic control of this trait and that variety selection could be useful in reducing acrylamide levels in rye products. High levels of nitrogen fertilisation were found to increase the accumulation of free asparagine, showing that excessive nitrogen application should be avoided in order not to exacerbate the problem of acrylamide formation. This effect of nitrogen was mitigated in two of the varieties by the application of sulphur.

## Highlights

► Acrylamide-forming potential is a key quality parameter for rye grain. ► Free asparagine level is main determinant of acrylamide-forming potential in rye. ► Rye varieties differ significantly in free asparagine concentration in the grain. ► High levels of nitrogen (N) increase the acrylamide-forming potential of rye. ► Sulphur reduces the effect of N on acrylamide-forming potential in some varieties.

## 1 Introduction

The formation of acrylamide, a probable human carcinogen (Friedman, 2003), in cooked, plant-derived foods, came to light eleven years ago (Tareke et al., 2002). Since then, much has been learnt about the mechanisms involved in its formation, and methods have been developed to reduce its presence in foods. These have been reviewed by Claus et al. (2008) and compiled in a ‘Toolbox’ produced by Food Drink Europe: (http://www.fooddrinkeurope.eu/uploads/publications_documents/Toolboxfinal260911.pdf).

The Maillard reaction is the primary route by which acrylamide forms (Mottram et al., 2002; Stadler et al., 2002). At high temperatures, amino groups, principally those of free amino acids, react with reducing sugars to produce a plethora of compounds that impart colour, flavour and aroma. Only when the last stage of the reaction involves asparagine does it produce acrylamide (Mottram et al., 2002; Stadler et al., 2002; Zyzak et al., 2003), with 3-aminopropionamide a possible transient intermediate (Granvogl and Schieberle, 2006). The Maillard reaction is responsible for many of the characteristics associated with fried, baked and roasted foods that consumers demand and that define particular products and brands. It is important that when steps are taken to mitigate acrylamide formation, the aspects of the reaction responsible for the production of colours, flavours and aromas are retained to ensure that the quality of the final product is not affected.

Potentially, one of the most effective methods of acrylamide mitigation would be to reduce the accumulation of acrylamide precursors in plant material used for food production. The identification of genetic and environmental factors that affect precursor content is, therefore, an important approach (Halford et al., 2012a) and has been the objective of studies performed on several major crops, including wheat and potatoes (Amrein et al., 2003; Halford et al., 2012b; Martinek et al., 2009; Muttucumaru et al., 2008; Shepherd et al., 2010). These studies have also shown differences in the relationship between precursor concentration and acrylamide formation, with free asparagine concentration correlating closely with acrylamide-forming potential in wheat but with a more complex picture emerging for potato, in which different studies have shown reducing sugar concentration, free asparagine concentration, or the ratio of free asparagine to other free amino acids to be the most important parameter (reviewed by Halford et al., 2012a).

A previous study on rye (Curtis et al., 2010) analysed the acrylamide precursor content of a number of old and modern rye varieties grown in different locations across Europe within the EU FP6 HEALTHGRAIN diversity programme (Ward et al., 2008). The authors concluded that free asparagine concentration was the main determinant of the level of acrylamide that forms in heated rye flour, and proposed that commercial varieties should be screened for low free asparagine concentration in the grain. In contrast to wheat, the study also showed correlations between sugar concentrations and acrylamide formation.

Sulphur (S) deficiency is the most important factor affecting acrylamide-forming potential in wheat grain, with free asparagine concentration in the grain of severely S-deprived wheat being up to 30-times higher than in the grain of wheat supplied with adequate S (Curtis et al., 2009; Granvogl et al., 2007; Muttucumaru et al., 2006). A similar response occurs in barley (Shewry et al., 1983). Nitrogen (N) has the opposite effect, suggesting that some plants use free asparagine as a N store (Lea et al., 2007), particularly when there is insufficient S available for the efficient synthesis of storage proteins (Shewry et al., 2001). S availability also causes changes in the distribution of free asparagine in wheat grain, with more accumulating in the endosperm (white flour) fraction of S-deficient grain and therefore affecting more products (Shewry et al., 2009).

In the present study, five current commercial varieties of rye were compared when grown in the same location at the same time, with nine different combinations of S and N application. Free amino acid and sugar concentrations in the grain were determined and related to the amount of acrylamide that formed in heated flour, with the aim of assessing differences in acrylamide-forming potential between the varieties, the relationship between precursor concentration and acrylamide formation and the importance of nutrient availability.

## 2 Materials and methods

### 2.1 Trial design

The trial used five rye varieties (Agronom, Askari, Festus, Fugato and Rotari) with seed provided by Saaten Union (Suffolk, UK). It was sown at the Rothamsted Farm site at Woburn, Bedfordshire, UK, which has a sandy loam soil with very poor nutrient retention (for example, soil sulphur (S) concentrations range from 0.5 to 1.8 mg kg^−1^) (Riley et al., 2002). S was applied as gypsum (calcium sulphate dihydrate) to give 0, 15 or 40 kg S ha^−1^, in combination with nitrogen (N) applied as ammonium nitrate to give 1, 100 or 200 kg N ha^−1^, resulting in nine different combinations of N and S. The trial was designed in a criss-cross layout, with the five varieties grown as main plots and nutrient treatments applied in rows and columns within each main plot. The main plots each comprised nine individual plots of 1.8 × 4 m and were separated by 3 m surrounds sown with sorghum to reduce cross-pollination between the varieties. Two blocks were used to provide biological replication. The trial was harvested in August 2010 and 5 g of grain from each plot was milled to fine, wholemeal flour for analysis.

### 2.2 Concentrations of free amino acids

Flour (0.5 g ± 0.005 g) was added to 10 mL of 0.01 N HCl and stirred for 15 min. The suspension was left to settle for 15 min at room temperature and an aliquot (1.5 mL) was centrifuged at 7200 *g* to produce a clear extract. Amino acids were derivatised using the EZ: Faast free amino acid kit (Phenomenex, Torrance, CA). Gas chromatography–mass spectrometry (GC–MS) analysis of the derivatised samples was carried out using an Agilent 6890 GC-5975-MS system (Agilent, Santa Clara, CA) in electron impact mode, as described by Elmore et al. (2005). An aliquot of the derivatised amino acid solution (1 μL) was injected at 250 °C in split mode (20:1) onto a Zebron ZB-AAA capillary column (10 m × 0.25 mm; 0.25 μm film thickness). The oven temperature was held at 110 °C for 1 min and then increased at 30 °C min^−1^ to 310 °C. The transfer line and ion source were maintained at 320 and 230 °C respectively; carrier gas flow rate was kept constant throughout the run at 1.1 mL min^−1^. Amino acid standards were provided with the EZ: Faast kit and were >99% pure (Phenomenex). Separate calibration curves were calculated for each amino acid. The standards were also used before, during and after the analysis of each batch of samples to check that the machine was running correctly. Analyses of the data were performed using the Agilent 5975 system data analysis software.

### 2.3 Analysis of sugars

The concentrations of reducing sugars and sucrose in the flour samples were measured using a method adapted from Curtis et al. (2010). Flour (0.5 g ± 0.005 g) was added to 10 mL methanol/water (50% v/v) containing 100 mg L^−1^ trehalose as an internal standard. The flour suspension was stirred for 15 min and left to settle for a further 15 min. An aliquot of the solution was then centrifuged at 7200 *g* to produce a clear extract; this was then diluted four-fold in water and filtered through a 0.2 μm syringe filter into a vial. A Dionex ion chromatography system with a CarboPac PA1 column and pulsed amperometric detection (Thermo, Waltham, MA) was used to analyse the sugar content of each sample. The injection volume was 25 μL and the eluent was 65% water and 35% 4 M NaOH at a flow rate of 1 mL min^−1^; at 11 min the eluent was changed to 50% water and 50% 4 M NaOH for the remainder of the run, the run ending at 18 min. The waveform of the pulsed amperometric detector was 400 ms at 0.1 V, 20 ms at −2.0 V, 10 ms at 0.6 V, and 60 ms at −0.15 V. Standard solutions of glucose, fructose, sucrose and maltose were used to produce calibration curves for quantification.

### 2.4 Analysis of acrylamide by liquid chromatography–mass spectrometry/mass spectrometry (LC–MS/MS)

Rye flour (0.5 g ± 0.005 g) was weighed into a 1 mL ampoule and heated at 160 °C for 20 min. The cooked flour was then added to 40 mL of water containing 2 μg of ^13^C_3_-acrylamide as an internal standard and shaken for 20 min. Centrifugation of the sample at 15 °C produced a clear extract, 2 mL of which were filtered through a 0.2 μm syringe filter into a vial for analysis. LC–MS/MS was performed using an Agilent 1200 high-performance liquid chromatography (HPLC) system connected to an Agilent 6410 triple quadrupole mass spectrometer. Separation was performed using a 100 × 3.0 mm Hypercarb column protected by a KrudKatcher (Phenomenex, Torrance, CA) and a pre-column (Hypercarb 10 mm × 3.0 mm 5 μm particle size; Thermo, Waltham, MA) with 0.1% formic acid as the mobile phase with a flow rate of 0.3 mL min^−1^. Most of the extract was not retained by the column and was eluted as waste before the acrylamide eluted at around 6 min. The transitions *m*/*z* 72→55 and *m*/*z* 72→27 were measured for acrylamide, and *m*/*z* 75→58 for ^13^C_3_-acrylamide.

### 2.5 Measurements of total grain N and S

Measurements of total grain N and S were made by the Analytical Unit of the Soil Science Department, Rothamsted Research. Total grain N was determined according to the Dumas digestion method, using a LECO CNS 2000 combustion analyser (Leco, Stockport, UK). Total S content was determined using an Accuris inductively coupled plasma optical emission spectrometer (ICP-OES) (Applied Research Laboratories, Vallaire, Ecublens, Switzerland; supplied by Thermo Optek, Crawley, UK) after the samples had been hydrolysed with a mixture of HNO_3_ and HClO_4_.

### 2.6 Statistical analysis

The GenStat^®^ statistical software (2010, Thirteenth Edition, VSN International Ltd, Hemel Hempstead, UK) was used to analyse the effects of variety, S and N on the concentrations of amino acids, sugars and acrylamide using analysis of variance (ANOVA). The least significant difference (LSD) at the 5% (*p* = 0.05) level, calculated from the standard error of the difference (SED) between means on the residual degrees of freedom (df) from the ANOVA, was used to make comparison of relevant means. GenStat^®^ was also used to perform a two-tailed test (F-test) of the (Pearson) correlations, *r*, between acrylamide measurements and the concentrations of its precursors. A multiple linear regression model for the acrylamide data was then fitted, using the method of forward selection of most significant (*p* < 0.05) explanatory variables. Inspection of residual plots revealed that no transformation of data was required for any of these analyses.

## 3 Results

### 3.1 Agronomic and varietal effects on free amino acid content of rye grain

**Fig. 1:**
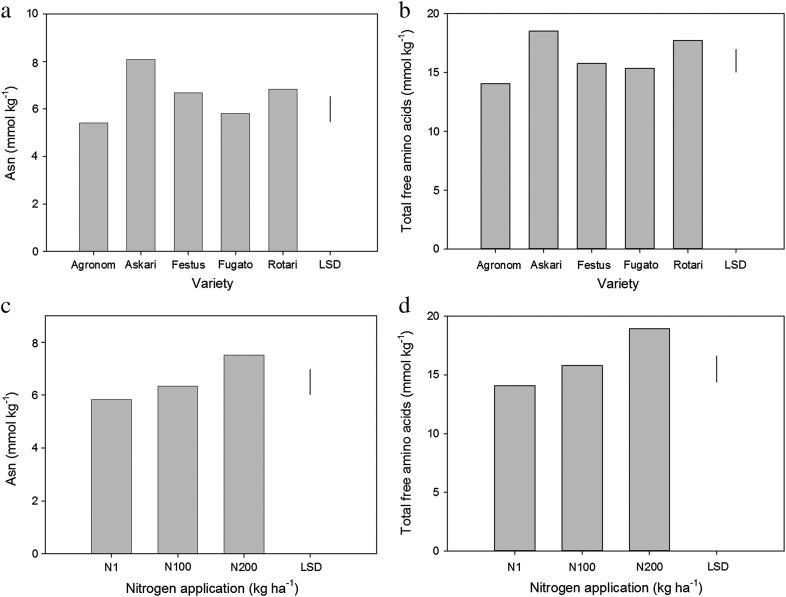
a. Mean free asparagine concentration (mmol kg^−1^) in grain of five commercially used rye varieties grown in 2009–2010 (*n* = 18). SED = 0.379 on 4 degrees of freedom (df). The least significant difference (LSD) at 5% = 1.051, and is indicated. b. Mean total free amino acid concentration (mmol kg^−1^) in grain of five rye varieties grown in 2009–2010 (*n* = 18). SED = 0.659 on 4 df. LSD (5%) = 1.830 c. Mean asparagine concentration (mmol kg^−1^) in grain of rye grown under different nitrogen fertilisation regimes (1, 100, 200 kg ha^−1^) (*n* = 30). SED = 0.428 on 10 df. LSD (5%) = 0.953. d. Mean total free amino acid concentration (mmol kg^−1^) in grain of rye grown under different nitrogen fertilisation regimes (1, 100, 200 kg ha^−1^) (*n* = 30). SED = 1.02 on 10 df. LSD (5%) = 2.272.

Five rye varieties (Agronom, Askari, Festus, Fugato and Rotari) were grown in a field trial with randomised design on sandy loam soil in Bedfordshire, UK, with nine different combinations of N (1, 100 or 200 kg ha^−1^) and S (0, 15 or 40 kg ha^−1^). Grain was harvested and milled to fine, wholemeal flour, which was analysed for free amino acid content by gas chromatography–mass spectrometry (GC–MS) after derivatisation. This method has been used previously to measure free amino acid concentrations in wheat and rye grain (Curtis et al., 2009, 2010; Granvogl et al., 2007; Muttucumaru et al., 2006). Note that it is not possible to measure arginine by this method. The data are presented in full in Supplementary File S1. Analysis of variance (ANOVA) showed that the different varieties accumulated significantly different amounts of free asparagine (*p* = 0.011, Fig. 1a), the lowest accumulators being Agronom and Fugato. Askari contained the highest concentration, accumulating almost 50% more free asparagine than Agronom (Askari = 8.08 mmol kg^−1^, Agronom = 5.42 mmol kg^−1^) and containing significantly more than any other variety in the trial (*p* < 0.05). ANOVA showed the effect of variety to be independent of any nutrient effects (*p* > 0.05), in other words the differences between varieties were not affected by N or S application. The varieties also showed significantly different concentrations of total free amino acids (*p* = 0.013, Fig. 1b).

The application of N to the plots was shown to be a significant factor affecting asparagine concentration (*p* = 0.008) (Fig. 1c). The concentration in grain grown at 1 kg ha^−1^ N was not statistically different (*p* > 0.05) from that of grain grown at 100 kg ha^−1^ N; however, the concentration in grain grown with 200 kg ha^−1^ N was significantly (*p* < 0.05) higher: 7.52 mmol kg^−1^ compared with 5.83 mmol kg^−1^ at 1 kg ha^−1^ N, representing a 29% increase in asparagine concentration from the 1 kg ha^−1^ N treatment to the 200 kg ha^−1^ treatment. All of the varieties responded in the same way; means were therefore calculated from all samples grown at each level of N application. There was also a significant (*p* = 0.002) effect of N on total free amino acids (Fig. 1d), with application at 200 kg ha^−1^ N giving a significant increase in free amino acid concentration compared with application at 1 or 100 kg ha^−1^ N (means (mmol kg^−1^): 14.19 at 1 kg ha^−1^ N; 15.90 at 100 kg ha^−1^ N; 19.04 at 200 kg ha^−1^ N), representing a 34% increase from the 1 kg ha^−1^ treatment to the 200 kg ha^−1^ treatment. In contrast, there was no significant effect of S application on free asparagine or total free amino acid accumulation (*p* > 0.05).

### 3.2 Agronomic and varietal effects on the accumulation of sugars in rye grain

The concentrations of sucrose and the reducing sugars, glucose, fructose and maltose, in the grain samples are presented in Supplementary File S1; means are shown in Table 1a. The sucrose and fructose concentrations showed marked differences from those of the varieties studied by Curtis et al. (2010). Sucrose was the most abundant sugar in both studies, but Curtis et al. (2010) reported sucrose concentrations of 22.81–38.85 mmol kg^−1^, whereas the varieties in the present study contained only 4.39–5.03 mmol kg^−1^. Fructose concentrations, on the other hand, were higher in the varieties analysed in this study: 2.88–4.37 mmol kg^−1^ compared with 0.26–1.47 mmol kg^−1^, making fructose the most abundant reducing sugar.

ANOVA showed that fructose, glucose and total reducing sugar concentration responded differently to N application in the five varieties, there being an interaction effect between variety and N (*p* = 0.019, 0.008 and 0.007 respectively) for all three variables. The relevant means are given in Table 1b. Agronom, for example, accumulated a higher concentration of reducing sugars at the intermediate N application level (100 kg ha^−1^ N) than at the extremes of N availability, whereas Askari responded to the intermediate N treatment with its lowest accumulation of reducing sugars. There was no effect of N on sucrose or maltose concentration (*p* > 0.05), and no significant effect of S on any of the sugars (*p* > 0.05) except sucrose, for which there was a marginally significant variety by S interaction (*p* = 0.045, Table 1c). This was largely due to the relatively high sucrose concentration for Rotari at 0 kg ha^−1^ S (compared with that of Agronom and Askari), and increasing S to 15 or 40 kg ha^−1^ resulted in a significant (*p* < 0.05) reduction in sucrose for this variety. There was an overall effect of variety for maltose (*p* = 0.006), Fugato and Rotari having greater concentrations than the other three varieties (Table 1d).

### 3.3 Acrylamide formation

**Fig. 2:**
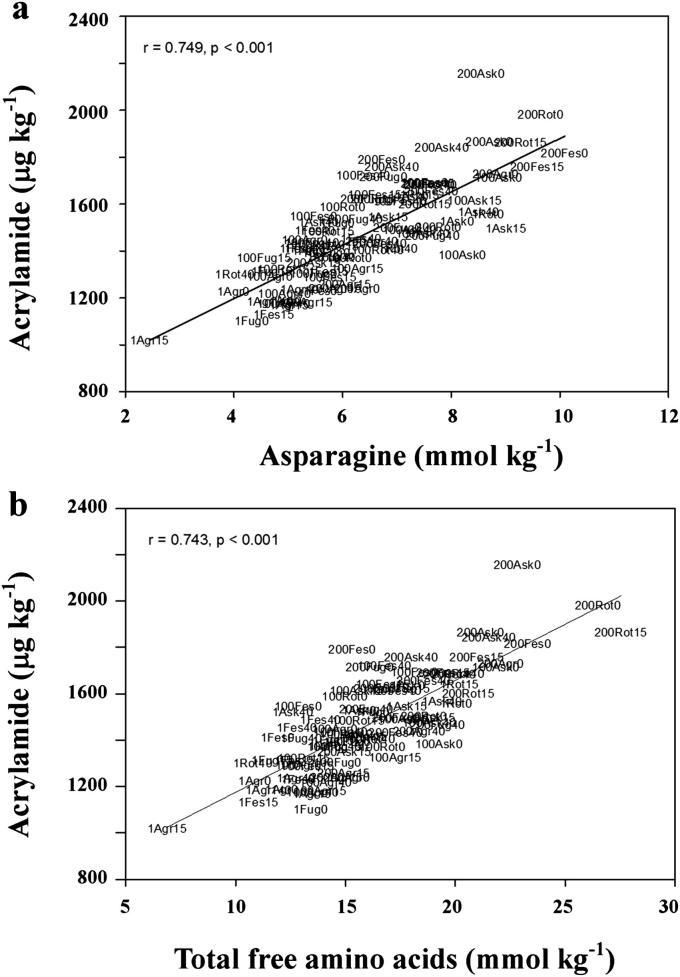
Acrylamide formed in flour heated at 160 °C (μg kg^−1^) plotted against free asparagine concentration (mmol kg^−1^ fresh weight) (top) and total free amino acid concentration (mmol kg^−1^ fresh weight) (bottom) for five commercial varieties of rye grown in 2009–2010. Each plot shows the Pearson correlation and the trend line. Plotted points are labels: The first number is the level of N (1, 100 or 200), then three letters indicate the variety name (**Agr**onom, **Ask**ari, **Fes**tus, **Fug**ato or **Rot**ari), and the last number is the level of S (0, 15 or 40). The plots show Pearson's correlation, *r*, with *p*-values.

Flour samples were heated at 160 °C for 20 min to allow the formation of acrylamide; the data are included in Supplementary File S1. There was a strong positive correlation between free asparagine concentration and acrylamide formation (*r* = 0.790, *p* < 0.001, *n* = 83) (Fig. 2). There was also a strong correlation between acrylamide formation and the concentration of total free amino acids (*r* = 0.743, *p* < 0.001, *n* = 83) (Fig. 2) and many individual free amino acids, namely aspartic acid, glutamic acid, isoleucine, leucine, proline and valine (graphs, Pearson's correlation and *p*-values given in Supplementary File S2). Other free amino acids can contribute to acrylamide formation by reacting with reducing sugars to give carbonyl compounds which react readily with asparagine to give acrylamide (Parker et al., 2012). Conversely, they may compete with asparagine for reaction with these carbonyl compounds in the later stages of the Maillard reaction (Halford et al., 2012a). However, any conclusions to be drawn from these correlations should be qualified because there was also a correlation between the concentrations of total free amino acids and free asparagine (asparagine was the most abundant free amino acid in the grain, accounting for 22–32% of the total) and between the concentrations of other individual free amino acids and free asparagine, namely aspartic acid, glutamic acid, proline, valine, threonine, glutamine, isoleucine, leucine, phenylalanine, serine and lysine (data not shown).

In contrast, there was no significant correlation between acrylamide formation and concentrations of glucose, fructose, maltose, total reducing sugars or sucrose (Supplementary File S2), showing free asparagine concentration to be the main determinant of acrylamide-forming potential in rye.

ANOVA showed that the overall effect of variety on acrylamide was significant (*p* = 0.005), as was the effect of N application (*p* = 0.002). There was only a marginal effect of S (*p* = 0.106) but the three factors of variety, N and S interacted to affect acrylamide concentration significantly (*p* = 0.026). The relevant means are given in Table 2 and show that the largest increases in acrylamide formation with increasing N occurred in Askari and Festus at 0 kg ha^−1^ S, the level increasing in Askari from 1579 μg kg^−1^ at 1 kg ha^−1^ N to 2009 μg kg^−1^ at 200 kg ha^−1^ N and in Festus from 1319 μg kg^−1^ at 1 kg ha^−1^ N to 1802 μg kg^−1^ at 200 kg ha^−1^ N. These represent increases of 27% and 36%, respectively. In both cases, the effect of N was mitigated by the effect of S, with application of 15 kg ha^−1^ S to Askari reducing acrylamide formation at 200 kg ha^−1^ N to 1321 μg kg^−1^ and application of 40 kg ha^−1^ S to Festus reducing the 200 kg ha^−1^ N acrylamide level to 1542 (LSD (5%) = 249.25). Surprisingly, the acrylamide level for Askari at 40 kg ha^−1^ S was not as low, and no significant effect of S was seen in the other varieties. Nevertheless, the data show that S application could reduce acrylamide-forming potential in the grain of some varieties of rye at high N application rates.

**Fig. 3:**
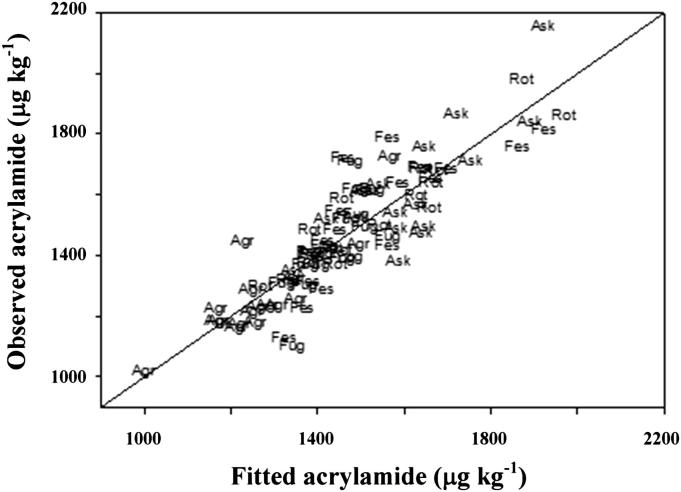
Observed acrylamide levels (μg kg^−1^) for five commercial varieties of rye grown in 2009–2010 plotted against levels (μg kg^−1^) predicted by the regression model: *Acrylamide* = *α* Asn + *β* Pro + *γ* Thr + *δ* Sucrose + Variety_*i*_ + *E*, where *α*, *β*, *γ* and *δ* are coefficients multiplying the asparagine, proline, threonine and sucrose concentrations, and where Variety_*i*_, for *i* = 1 (Agronom), 2 (Askari), 3 (Festus), 4 (Fugato) and 5 (Rotari), are five additive effects for the five varieties and *E* is the error term. The line indicates the 1:1 relationship. Plotted points are labels: The three letters indicate the variety name (**Agr**onom, **Ask**ari, **Fes**tus, **Fug**ato or **Rot**ari), noting that N and S factors were not significant in the model.

A model for the acrylamide data was derived using regression analysis. This showed that the concentration of asparagine in the grain explained most of the variance in acrylamide formation (61.8%). Two other amino acids contributed to acrylamide variance: proline (6.7%) and threonine (1.2%). Sucrose was also found to contribute marginally to the explanation of variance (1.4%) and there was an additional effect of variety (5.8%) which was not explained by amino acids or sugars, possibly indicating effects of other aspects of grain chemistry. The best model was:

$$\text{Acrylamide}=\alpha \;\text{Asn}+\beta \;\text{Pro}+\gamma \;\text{Thr}+\delta \;\text{Sucrose}+{\text{Variety}}_{i}+E\text{,}$$

where *α*, *β*, *γ* and *δ* are coefficients multiplying the asparagine, proline, threonine and sucrose concentrations, where Variety_*i*_, for *i* = 1 (Agronom), 2 (Askari), 3 (Festus), 4 (Fugato) and 5 (Rotari), are five additive effects for the five varieties and where *E* is the error term. The model accounts for 75.6% (*R*^2^) of the variance in the observed data and uses only nine parameters. The standard error of observations given the model is 106 (μg kg^−1^) on 76 df. It should be noted, however, that regression analysis is a purely statistical exercise, and there is no explanation for the contribution of threonine or proline to the variance from what is known about the chemistry of acrylamide formation. Proline has been shown to inhibit acrylamide formation rather than increase it (Koutsidis et al., 2009) but that would be unlikely to occur when proline is present at much lower concentrations than asparagine, as is the case here. Similarly, while sucrose can take part in the Maillard reaction if it is first hydrolysed (De Vleeschouwer et al., 2009), reducing sugars participate much more readily. Nevertheless, the quality of the fit of the model to the data (Fig. 3) is shown as closely following a 1:1 line. The estimates of parameters in the model are given in Supplementary File S2.

### 3.4 Effect of fertiliser treatment on the N and S content of rye grain

The analysis of total grain N (Table 3a) showed a significant effect of variety (*p* = 0.031), accounted for by a higher N content of Rotari compared with the other varieties. The mean % N of Rotari (1.77) was 9% higher than that of Festus, which contained the lowest % N (1.62). There was also a significant interaction between the N and S treatments (*p* = 0.010). The relevant means are given in Table 3b and show that, although increasing N application consistently resulted in increased N content of the grain, the availability of S became important at the highest N treatment. Hence grain grown with N applied at a rate of 200 kg ha^−1^ and S at a rate of 0 kg ha^−1^ had a lower N content (1.96%) than that grown with 15 kg ha^−1^ S (2.10%) or 40 kg ha^−1^ S (2.18%). Since there was no effect of S on total free amino acid concentration, the increased accumulation of N in grain grown under high N and S application suggested increased protein content. Conversely, the plants responded to lower S availability by reducing the N content of the grain rather than accumulating more free asparagine.

The analysis of grain S also showed an interaction between the S and N treatments (*p* < 0.001). The relevant means are given in Table 3c and show that, at all three N applications, grain S increased when S application was increased from 0 to 15 kg ha^−1^, but only at a N application of 200 kg ha^−1^ did increasing the S to 40 kg ha^−1^ produce another significant (*p* < 0.05) increase in grain S (1373–1442 ppm). At a given level of S application, increasing N application increased the S content of the grain, although at 0 kg ha^−1^ S there was no significant (*p* > 0.05) increase in grain S when N was increased from 100 to 200 kg ha^−1^. There was no change in the abundance of free methionine with increasing grain S (Supplementary File S1), suggesting that the S was contained within proteins, although the quantity of cysteine, which also contains S, could not be determined. This may explain why there was a relationship between increasing N and increasing S, as at high N availability there would be more resources for the synthesis of S-rich storage proteins. The lack of increase in grain S when S was not applied and N was high may be due to insufficient S being available to produce proteins, unlike at the other levels of S availability.

There was also an interaction between N application and variety on the S content of the grain (*p* = 0.022). The relevant means are given in Table 3d. This was accounted for by a different response of Festus to the different N applications. When N application was increased from 100 to 200 kg ha^−1^, Festus did not accumulate more S in the grain, whereas the other varieties did. Festus had the lowest S content at 100 kg ha^−1^ N (1138 ppm) of all the varieties, and also had lowest S content at 200 kg ha^−1^ N (1191 ppm) (Table 3d).

## 4 Discussion

The main aim of this study was to compare the acrylamide-forming potential of five important commercial rye varieties used for crispbread manufacture, and relate this to precursor concentration. Agronom and Fugato were identified as varieties with relatively low acrylamide-forming potential and the study showed free asparagine concentration to be the key factor in determining acrylamide formation in heated rye flour. However, asparagine levels correlated closely with total free amino acid concentrations and this will have to be taken into consideration if free asparagine levels are to be reduced through breeding because of the importance of other free amino acids for the production of flavour and colour compounds during baking.

Modelling also indicated a weak influence of sucrose on acrylamide formation, but no significant correlation was evident with reducing sugars. This contrasted with a previous study which found free asparagine to be the most important factor but also showed correlations between acrylamide formation and sugar concentrations (Curtis et al., 2010). The varieties analysed in that study had much higher sugar concentrations than those studied here, which may explain the difference. Indeed, the differences in concentrations of sugars between the varieties studied here and those studied by Curtis et al. (2010) were notable, particularly for sucrose, which ranged from 22.81 to 38.85 mmol kg^−1^ in the Curtis et al. (2010) study but only from 4.39 to 5.03 mmol kg^–1^ here. However, there is surprisingly little information on sugar concentrations in rye or other cereal grain (Halford et al., 2011), so it is impossible to say what the ‘normal’ range is. Curtis et al. (2010) analysed a diverse selection of varieties, including some that are no longer used in cultivation, while those studied here were modern, elite commercial varieties. The grain analysed by Curtis et al. (2010) was also produced in France, Hungary, Poland and the UK over several growing seasons, while the grain analysed in this study was from a single location and year, and sugar concentrations in plants can change greatly in response to environmental conditions (Halford et al., 2011). Furthermore, the range over the two studies, from 4.39 to 38.85 mmol kg^–1^, is actually narrower than has been reported for maize (12.91–89.60 mmol kg^–1^; Harrigan et al., 2007) or rice (15.10–58.60 mmol kg^–1^; Smyth et al., 1986).

High levels of nitrogen (N) application (200 kg ha^−1^) significantly increased acrylamide formation during heating. The current recommendation for N fertilisation (Department for Environment, Food and Rural Affairs, Great Britain, 2010) advises application of 70 kg ha^−1^ to rye grown in the conditions of the Woburn trial site. This recommendation increases to a maximum of 160 kg ha^−1^ for crops grown on sandy soils in areas with high annual rainfall, as these sites experience leaching of much of the N applied. These recommendations are designed to reduce the environmental impact and cost of over-fertilising crops, but the effect of high N application on acrylamide-forming potential is another good reason to avoid over-fertilisation with N. There was no direct effect of S, in contrast with wheat, in which S deprivation leads to highly elevated acrylamide-forming potential (Curtis et al., 2009; Granvogl et al., 2007; Muttucumaru et al., 2006). The response seen in wheat may be attributed, at least in part, to increased expression of asparagine synthetase (Byrne et al., 2012) and the use of free asparagine as an alternative grain N store when wheat is unable to synthesise storage proteins (Curtis et al., 2009). Rye may be better able to scavenge available S from the soil than wheat (Bushuk, 2001), but may also respond differently to S deficiency. At low levels of S application, grain N content was reduced, even when plenty of N was available, while free amino acid concentrations were not affected. In other words, rye appeared to accumulate less N in the grain when it had insufficient S available, rather than use free asparagine as an alternative N store.

Although S application had no direct effect on acrylamide-forming potential, it did mitigate the effect of high N application in two of the varieties, Askari and Festus. This is the first evidence that S application could be beneficial in reducing the acrylamide-forming potential of rye. However, the effect was variety-dependent and only seen with high N application.

## Figures and Tables

**Table 1a:** Sugar concentrations (mmol kg^−1^) in the grain of five commercial varieties of rye. Mean total and individual sugar concentrations (mmol kg^−1^) in the grain (*n* = 18) are given with standard errors in parenthesis, and the percentage of total sugars they represent.

	Agronom	Askari	Festus	Fugato	Rotari
Total sugars	10.44 (0.52)	9.92 (0.59)	11.22 (0.44)	12.77 (0.55)	12.13 (0.55)
Glucose	0.90 (8.6%) (0.06)	0.91 (9.2%) (0.06)	1.13 (10.1%) (0.05)	1.19 (9.3%) (0.06)	1.08 (8.9%) (0.05)
Maltose	1.80 (17.2%) (0.10)	1.64 (16.5%) (0.10)	1.50 (13.4%) (0.10)	2.25 (17.6%) (0.13)	2.42 (20%) (0.14)
Fructose	3.35 (32.1%) (0.36)	2.88 (29.0%) (0.36)	3.74 (33.3%) (0.26)	4.37 (34.2%) (0.33)	3.60 (29.7%) (0.26)
Sucrose	4.39 (42.0%) (0.13)	4.49 (45.3%) (0.18)	4.85 (43.2%) (0.15)	4.96 (38.8%) (0.16)	5.03 (41.5%) (0.19)

**Table 1b:** Mean concentrations (mmol kg^−1^) of fructose, glucose and total reducing sugars (TRS) in the grain of the five rye varieties grown under different nitrogen (N) fertilisation regimes (1, 100, 200 kg ha^−1^) in 2009–2010 (*n* = 6). There were significant interactions between variety and N for fructose (*p* = 0.019, *F*-test), glucose (*p* = 0.008) and total reducing sugar content (*p* = 0.007).

Variety	N (kg ha^−1^)	Sugar concentration (mmol kg^−1^)		
		Fructose	Glucose	TRS
Agronom	1	2.51	0.71	4.88
	100	4.70	1.14	7.77
	200	2.86	0.85	5.51
Askari	1	3.78	1.06	6.51
	100	1.95	0.69	4.20
	200	2.89	0.99	5.57
Festus	1	3.38	0.98	5.87
	100	4.17	1.25	6.93
	200	3.66	1.16	6.30
Fugato	1	4.46	1.33	7.97
	100	5.55	1.30	9.19
	200	3.09	0.93	6.27
Rotari	1	3.31	1.00	6.87
	100	3.45	0.99	6.67
	200	4.05	1.25	7.77

Standard error of the difference (SED) for comparisons of fructose: 1. Means with same variety (different N): SED = 0.643 on 10 df; LSD (5%) = 1.433, 2. All other comparisons: SED = 0.642 on 13 df; LSD (5%) = 1.377. Standard error of the difference (SED) for comparisons of glucose: 1. Means with same variety (different N): SED = 0.127 on 10 df; LSD (5%) = 0.282, 2. All other comparisons: SED = 0.139 on 12 df; LSD (5%) = 0.302. Standard error of the difference (SED) for comparisons of total reducing sugars: 1. Means with same variety (different N): SED = 0.725 on 10 df; LSD (5%) = 1.615, 2. All other comparisons: SED = 0.779 on 12 df; LSD (5%) = 1.684.

**Table 1c:** Mean concentrations (mmol kg^−1^) of sucrose in the grain of five rye varieties grown under different sulphur (S) fertilization regimes (0, 15, 40 kg ha^−1^) in 2009–2010 (*n* = 6). There was a significant interaction between variety and S (*p* = 0.045).

Variety	S (kg ha^−1^)	Sucrose content (mmol kg^−1^)
Agronom	0	4.34
	15	4.18
	40	4.64
Askari	0	4.39
	15	4.12
	40	4.97
Festus	0	4.80
	15	5.23
	40	4.52
Fugato	0	4.78
	15	5.33
	40	4.78
Rotari	0	5.56
	15	4.69
	40	4.85

Standard error of the difference (SED) for comparisons: 1. Means with same variety (different S): SED = 0.326 on 10 df; LSD (5%) = 0.725, 2. All other comparisons: SED = 0.442 on 8 df; LSD (5%) = 1.005.

**Table 3a:** Mean total N (% dry weight) for five varieties of rye grown in 2009–2010 under all conditions (*n* = 18). There was a significant effect of variety (*p* = 0.031).

Variety	N (% dry weight)	Variety	N (% dry weight)
Agronom	1.67	Fugato	1.66
Askari	1.67	Rotari	1.77
Festus	1.62		

Standard error of the difference (SED) for comparisons: SED = 0.028 on 4 df, LSD (5%) = 0.078.

**Table 3b:** Mean N content (% dry weight) of grain from five varieties of rye grown in 2009–2010 under different nutrient application regimes (*n* = 10). N content was affected significantly (*p* = 0.010) by an interaction between N and S.

N (kg ha^−1^)	S (kg ha^−1^)		
	0	15	40
1	1.34	1.32	1.31
100	1.61	1.64	1.63
200	1.96	2.10	2.18

Standard error of the difference (SED) for comparisons: 1. Means with same N level (different S): SED = 0.046 on 27 df; LSD (5%) = 0.094. 2. Means with same S level (different N): SED = 0.053 on 24 df; LSD (5%) = 0.110. 3. All other comparisons: SED = 0.054 on 27 df; LSD (5%) = 0.111.

**Table 3c:** Mean S content (ppm dry weight) of grain from five varieties of rye grown in 2009–2010 under different nutrient application regimes (*n* = 10). S content was affected significantly (*p* < 0.001) by an interaction between N and S.

N (kg ha^−1^)	S (kg ha^−1^)		
	0	15	40
1	1042	1096	1108
100	1105	1243	1259
200	1130	1373	1442

Standard error of the difference (SED) for comparisons: 1. Means with same N level (different S): SED = 25.9 on 27 df; LSD (5%) = 53.1. 2. Means with same S level (different N): SED = 23.2 on 26 df; LSD (5%) = 47.7. 3. All other comparisons: SED = 24.2 on 32 df; LSD (5%) = 49.3.

**Table 1d:** Mean concentrations (mmol kg^−1^) of maltose in the grain of five rye varieties (*n* = 18). There were significant difference between varieties (*p* = 0.006).

Variety	Maltose (mmol kg^−1^)	Variety	Maltose (mmol kg^−1^)
Agronom	1.78	Fugato	2.25
Askari	1.64	Rotari	2.42
Festus	1.50		

SED = 0.1212 on 4 df; LSD (5%) = 0.3365.

**Table 2:** Means (μg kg^−1^) for acrylamide formation in heated flour for the significant (*p* = 0.026) interaction between variety, N and S in five commercial varieties of rye grown in 2009–2010.

Variety	N (kg ha^−1^)	S (kg ha^−1^)		
		0	15	40
Agronom	1	1205	1092	1209
	100	1368	1251	1195
	200	1483	1248	1348
Askari	1	1579	1519	1543
	100	1547	1497	1481
	200	2009	1321	1798
Festus	1	1319	1221	1469
	100	1618	1464	1665
	200	1802	1722	1542
Fugato	1	1309	1355	1389
	100	1347	1462	1327
	200	1604	1620	1499
Rotari	1	1465	1527	1354
	100	1478	1401	1414
	200	1741	1730	1683

Standard error of the difference (SED) for comparisons: 1. Means with same variety and N level (different S): SED = 120.2 on 22 df; LSD (5%) = 249.3. 2. Means with same variety and S level (different N): SED = 136.9 on 19 df; LSD (5%) = 284.4. 3. Means with same variety (different N and S): SED = 154.8 on 24 df; LSD (5%) = 319.1. 4. All other comparisons: SED = 139.0 on 32 df; LSD (5%) = 283.0.

**Table 3d:** Nitrogen (N) and sulphur (S) content of grain for five commercial varieties of rye grown in 2009–2010. Mean S content (ppm dry weight) of grain from each variety grown under different nutrient application regimes (*n* = 6). S content was affected significantly (*p* = 0.022) by an interaction between variety, N and S.

Variety	N (kg ha^−1^)		
	1	100	200
Agronom	1084	1261	1415
Askari	1149	1222	1317
Festus	1012	1138	1191
Fugato	1055	1153	1296
Rotari	1109	1240	1357

Standard error of the difference (SED) for comparisons: 1. Means with same variety level (different N): SED = 24.77 on 10 df; LSD (5%) = 55.20. 2. All other comparisons: SED = 40.58 on 6 df; LSD (5%) = 96.58.
