# Supplementary material for: Effects of variety and nutrient availability on the acrylamide-forming potential of rye grain
Source: J Cereal Sci. 2013 May;57(3):463–70. doi: 10.1016/j.jcs.2013.02.001 (PMC3688320; doi:10.1016/j.jcs.2013.02.001)
Supplement: Supplementary file 2 [file mmc2.docx]

**Supplementary File S2**

Acrylamide formed in flour heated at 160°C (µg kg^-1^) plotted against free amino acid concentrations (mmol kg^-1^ fresh weight): Aspartate, glutamate, isoleucine, leucine, proline and valine, as indicated, for five commercial varieties of rye grown in 2009-2010. Each plot shows the Pearson correlation and the trend line. Plotted points are labels: the first number is the level of N (1, 100 or 200), then three letters indicate the variety name (**Agr**onom, **Ask**ari, **Fes**tus, **Fug**ato or **Rot**ari), and the last number is the level of S (0, 15 or 40). The plots show Pearson’s correlation, *r*, with p-values.


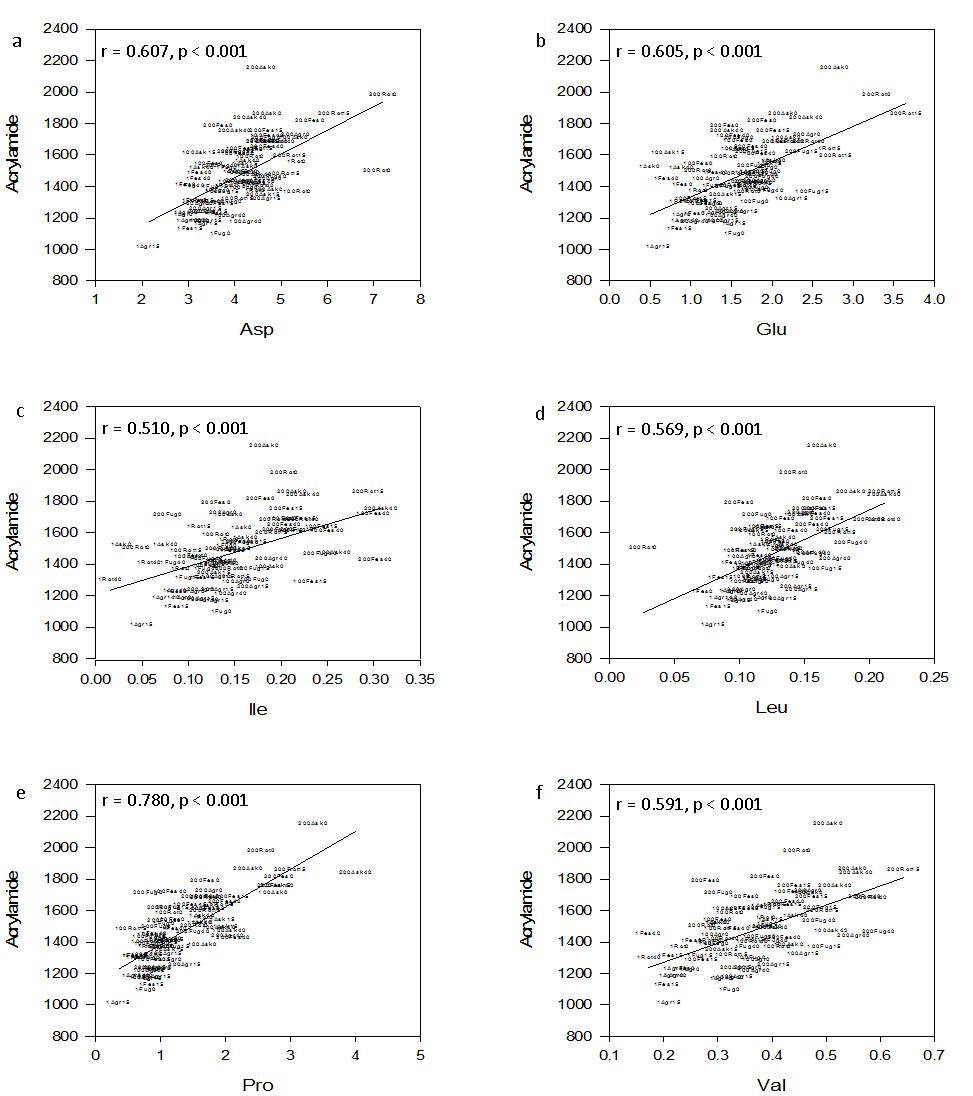


Acrylamide formed in flour heated at 160°C (µg kg^-1^) plotted against sugar concentrations (mmol kg^-1^) a) Glucose, b) Fructose, c) Maltose, d) Total reducing sugars, e) Sucrose, for five commercial varieties of rye grown in 2009-2010. Plotted points are labels: the first number is the level of N (1, 100 or 200), then three letters indicate the variety name (**Agr**onom, **Ask**ari, **Fes**tus, **Fug**ato or **Rot**ari), and the last number is the level of S (0, 15 or 40). There were no significant (p < 0.05, F-test) correlations.

Parametric details of the model fitted to acrylamide data from five commercial varieties of rye grown in 2009-2010. The model was Acrylamide = *α* Asn + *β* Pro + *γ* Thr + *δ* Sucrose + Variety_i_ + *E*, where *α*, *β*, and *γ* and *δ* are coefficients multiplying the asparagine, proline, threonine and sucrose quantities, where *Variety_i_*, for *i* = 1,…, 5 are five additive effects for the five varieties and *E* is the error term. R^2^ = 75.6%, s^2^ = 11206 on 76 df.

| **Parameter** | **Estimate (SE)** |
| --- | --- |
| *α* | 39.7 (13.8) |
| *β* | 156.8 (29.1) |
| *γ* | 536 (209) |
| *δ* | 20.4 (18.6) |
| *Agronom* | 751.2 (90.5) |
| *Askari* | 824.7 (99.1) |
| *Festus* | 877.0 (100.0) |
| *Fugato* | 893.2 (99.9) |
| *Rotari* | 887.0 (104.0) |
